# Cold Response Transcriptome Analysis of the Alternative Splicing Events Induced by the Cold Stress in *D. catenatum*

**DOI:** 10.3390/ijms23020981

**Published:** 2022-01-17

**Authors:** Yan Zheng, Landi Luo, Qian Chen, Danni Yang, Yuqiang Gong, Ya Yang, Xiangshi Qin, Yuhua Wang, Xiangxiang Kong, Yongping Yang

**Affiliations:** 1 The Germplasm Bank of Wild Species, Kunming Institute of Botany, Chinese Academy of Sciences, Kunming 650201, China; zhengyan@mail.kib.ac.cn (Y.Z.); chenqian@mail.kib.ac.cn (Q.C.); yangdanni@mail.kib.ac.cn (D.Y.); gyq415@126.com (Y.G.); yangya@mail.kib.ac.cn (Y.Y.); qinxiangshi@mail.kib.ac.cn (X.Q.); wangyuhua@mail.kib.ac.cn (Y.W.); 2 CAS Laboratory of Tropical Plant Resources and Sustainable Use, Xishuangbanna Tropical Botanical Garden, Chinese Academy of Sciences, Menglun, Mengla 666303, China; luolandi@xtbg.ac.cn; 3 University of Chinese Academy of Sciences, Beijing 100049, China; 4 College of Horticulture and Landscape, Yunnan Agricultural University, Kunming 650500, China

**Keywords:** *Dendrobium catenatum Lindl*, alternative splicing, cold stress, *DcCBP20*

## Abstract

*Dendrobium catenatum Lindl* is a valuable medicinal herb and gardening plant due to its ornamental value and special medical value. Low temperature is a major bottleneck restricting *D. catenatum* expansion towards the north, which influences the quality and yield of *D. catenatum*. In this study, we analysed the cold response of *D. catenatum* by RNA-Seq. A total of 4302 differentially expressed genes were detected under cold stress, which were mainly linked to protein kinase activity, membrane transport and the glycan biosynthesis and metabolism pathway. We also identified 4005 differential alternative events in 2319 genes significantly regulated by cold stress. Exon skipping and intron retention were the most common alternative splicing isoforms. Numerous genes were identified that differentially modulated under cold stress, including cold-induced transcription factors and splicing factors mediated by AS (alternative splicing). GO enrichment analysis found that differentially alternatively spliced genes without differential expression levels were related to RNA/mRNA processing and spliceosomes. DAS (differentially alternative splicing) genes with different expression levels were mainly enriched in protein kinase activity, plasma membrane and cellular response to stimulus. We further identified and cloned *DcCBP20* in *D. catenatum*; we found that *DcCBP20* promotes the generation of alternative splicing variants in cold-induced genes under cold stress via genetic experiments and RT–PCR. Taken together, our results identify the main cold-response pathways and alternative splicing events in *D. catenatum* responding to cold treatment and that *DcCBP20* of *D. catenatum* get involved in regulating the AS and gene expression of cold-induced genes during this process. Our study will contribute to understanding the role of AS genes in regulating the cold stress response in *D. catenatum*.

## 1. Introduction

Cold stress is a significant environmental factor that has an adverse impact on plant growth and development, as well as plant spatial distribution and crop productivity [1]. Plants that originate in a temperate area, like winter wheat, *Arabidopsis*, barley, and oilseed rape, have a high chilling resistance and can improve their freezing tolerance when exposed to cold but not freezing temperatures for a period. However, many crops, including rice, tomato, soybean and cotton, are susceptible to cold stress and lack of cold acclimation mechanisms, causing them to grow exclusively in tropical or subtropical regions [2,3]. At both the physiological and molecular levels, plants have developed a series of mechanisms to acclimatize to cold stress. When plants are exposed to nonfatal low temperatures for a period, they establish an enhanced ability to resist subsequent cold stress, which is termed cold acclimation [4,5]. During this process, cold signaling changes the fluidity of cellular membranes and calcium (Ca^2+^) influx, which is a critical process to trigger downstream cold-response gene expression [6,7].

The most typical cold response pathway is the CBF/DREB1-COR (C-Repeat Binding Factor/Dehydration-Responsive Element-Binding Protein 1-Cold Regulated)-dependent transcriptional regulatory pathway, which plays a key role in cold resistance. CBF/DREB1 can bind to cis-elements in the promoters of COR genes and activate their expression under cold stress, thereby increasing plant cold tolerance. COR is an important element that can protect plants from cold damage by encoding osmolyte and cryoprotective proteins and inducing their expression [8,9,10,11]. In addition, posttranscriptional and posttranslational regulatory mechanisms have been shown to be important for cold responses. Previous studies reported that ubiquitination, sumoylation, and phosphorylation mediated by protein kinases play major roles in responding to the cold tolerance regulatory pathway in plants. Many protein kinase families have been proven to play crucial roles in responding to low temperature, including MAPKs (Mitogen-Activated Protein Kinases) and CRLK1 (Calcium/Calmodulin-Regulated Receptor-Like Kinase 1) [12,13]. The most recent study revealed that phytohormones are involved in regulating *CBF* expression and plant cold tolerance, such as BRs (brassinosteroids), JA (jasmonic acid) and ethylene [14,15,16]. Emerging evidences indicate that many clock-related transcription factors are closely associated with the cold stress response [17].

Alternative splicing (AS) is a widespread process that a single gene can create diversity mRNA splicing variant via different splicing methods. This process can produce numerous kinds of new transcript and protein variants [18]. There are five subtypes of transcript variations resulting from AS: IR (intron retention), A3SS (alternative 3′ splice sites), A5SS (alternative 5′ splice sites), ES (exon skipping), and MXE (mutually exclusive exons). IR is the most characterized AS type in plants; indeed, approximately 40% to 60% of intron-containing genes in plants are estimated to exhibit one or more types of AS forms [19,20]. Previous studies have indicated that AS is involved in various plant development periods and stress responses, including flowering regulation, heat, salt, and other biotic stresses [21]. For example, more than 6000 genes undergo AS events in *Arabidopsis* when exposed to salt stress [22]. Low temperature can also cause AS events. According to previous studies, twenty-seven percent of chilling-responsive transcripts are alternatively spliced [23]. The spliceosome is a crucial component for AS generation and comprises numerous Ser/Arg-rich (SR) proteins and small nuclear ribonucleoproteins (snRNPs) [24]. Moreover, previous studies have shown that the plant nuclear cap-binding complex (CBC), including two subunits (CBP20 and CBP80), can affect the splicing of plant pre-mRNAs. The nuclear cap-binding complex (CBC), which functions as a key mediator in 7 mG on mRNA, takes part in eukaryotic gene expression. CBC is an essential part of several gene expression events, including splicing, transcription and translation. The nuclear CBC is thought to play a crucial role in microRNA biogenesis, pre-mRNA alternative splicing, flowering and responding to environmental stress [25,26,27]. Several reports have revealed that two subunits of CBC in *Arabidopsis*, *AtCBP20* and *AtCBP80,* are involved in abiotic stresses. One study showed that *AtCBP20* and *AtCBP80* are involved in salt stress tolerance by modulating the alternative splicing of genes involved in sugar metabolism. Another study provided evidence that CBP20 participates in fine-tuning splicing factors when exposed to salt stress conditions by interacting with SR45a [28,29]. However, the molecular mechanism underlying the role of CBP20 in the crosstalk between AS regulators and cold responses remains unclear.

*Dendrobium catenatum Lindl*, an important perennial epiphytic orchid, is mainly distributed in the southern and western mountain ranges of China, such as northwestern Guangxi, Sichuan and southeastern Yunnan Provinces. *D. catenatum* has great economic values due to its uses in herbal medicine and ornamental gardens. In addition, it has high research values because of its flower morphology and multiple secondary metabolites, including antitumour and immunomodulatory effects [30]. Large and persisting market demands have led to the overharvesting of wild *D. catenatum*. Therefore, it is necessary to expand the planting region of *D. catenatum* to improve yield. Generally, temperatures between 10 °C and 25 °C are the best fit for orchid plants, such as *D. catenatum* [31]. Most *D. catenatum* cultivars have been adapted to warmer southern climates, so they are vulnerable to damage by chilling stress when transplanted in regions north of their local habitat areas. Low temperature is a crucial bottleneck that restricts the spread of plants northwards. A previous study found that the aerial part of *D. catenatum* is very sensitive to chilling stress, and the plant leaf surface is pitted and discoloured, usually followed by wilting and browning, which seriously affects the normal growth rate of plants [32]. In addition, low temperature can bring out metabolic and physiological changes that are bad for the yield and quality of the plants. It has been reported that the contents of polysaccharides, SOD enzyme activity and alkaloids in chilling-tolerant *D. catenatum* cultivars are higher than those in chilling-sensitive cultivars [33]. Thus, investigating how plants react to cold stress will provide invaluable knowledge and genetic information for improving cold-resistant tolerance in *D. catenatum*. However, the specific regulatory mechanism of chilling responses in *D. catenatum* remains unclear. It has been shown that AS networks participate in the cold response as central coordinators, but the impact of AS on cold responses in *D. catenatum* was previously unexplored.

In this study, we analysed gene expression changes and identified the differential alternative splicing isoforms of cold-induced genes in *D. catenatum* with and without chilling stress by RNA-seq. We further identified the *DcCBP20* gene of *D. catenatum* and found that *DcCBP20* can alter alternative splicing variants when exposed to cold stress. This study will contribute to better understanding the cold-responsive pathways and the functions of AS in *D. catenatum* in response to chilling stress and is the first to report that *DcCBP20* contributes to chilling tolerance in *D. catenatum*.

## 2. Results

### 2.1. Damage to D. catenatum under Cold Stress

**Figure 1:**
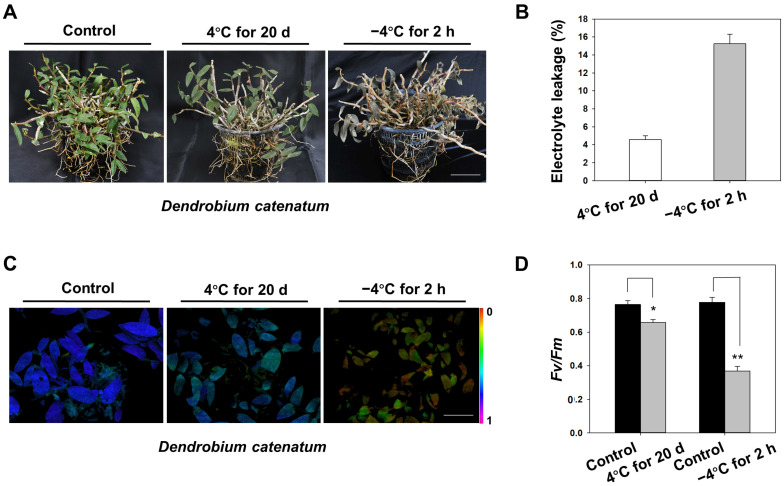
Analysis of cold resistance of *D. catenatum*. (**A**) Representative phenotypes of plants under chilling (4 °C for 20 d) and freezing (−4 °C for 2 h) conditions (bar = 5 cm). (**B**) Electrolyte leakage (EL) of the leaves of *D. catenatum* after chilling (4 °C for one week) and freezing (−4 °C for 2 h) stress. (**C**) Chl fluorescence imaging of *D. catenatum* after chilling and freezing stress (bar = 2 cm). (**D**) *Fv*/*Fm* ratios of *D. catenatum* after chilling and freezing stress. Data are the mean ± SD of three independent biological replicates. Statistical analyses were performed using a Student’s *t* test. **, *p* < 0.01; and *, *p* < 0.05.

To investigate the cold tolerance of *D. catenatum*, plants were subjected to low temperature. The morphological performance of *D. catenatum* with and without cold stress was significantly different. Compared to the plants under normal growth conditions, *D. catenatum* exposed to 4 °C or −4 °C showed typical cold injury symptoms. Chilling stress limited its normal growth flush; the leaves of chilling-treated plants exhibited surface pitting; the stem showed wilting and browning symptoms. For the freezing plants, the foliage became desiccated; plant sections died (Figure 1A). Consistent with these significant cold injury symptoms, we found that the Chl fluorescence in chilled *D. catenatum* plants was repressed after chilling treatment, with a lower *Fv*/*Fm* level compared to the control plants. For the freezing experiment, the Chl fluorescence of frozen plants was significantly prohibited compared to that of the chilled and normal plants, and the relative electrolyte leakage of frozen plants was significantly increased compared to chilled plants (Figure 1B−D). These results indicate that *D. catenatum* is likely a kind of cold-sensitive plant, and freezing stress can seriously affect the normal growth of *D. catenatum* and eventually cause death.

### 2.2. Transcriptional Changes Related to Chilling Stress in D. catenatum

To further investigate the molecular basis of the *D. catenatum* in response to cold stress, we performed the RNA-seq method of the total RNA isolated in the leaves of *D. catenatum* planted at 25 °C and transferred to 4 °C for 12 h. A total of 22,687 expressed genes with 2830 novel genes were identified referring to the *Dendrobium catenatum Lindl* genome sequence (http://orchidbase.itps.ncku.edu.tw/est/Dendrobium_2019.aspx, accessed on 5 June 2021) (Figure S1; Tables S1–S4). According to principal component analysis (PCA) and Pearson correlation analysis among different samples, the replicates of the plant samples with and without cold treatment were clustered closely independently, indicating that the results were highly reproducible with high quality (Figures S2 and S3).

**Figure 2:**
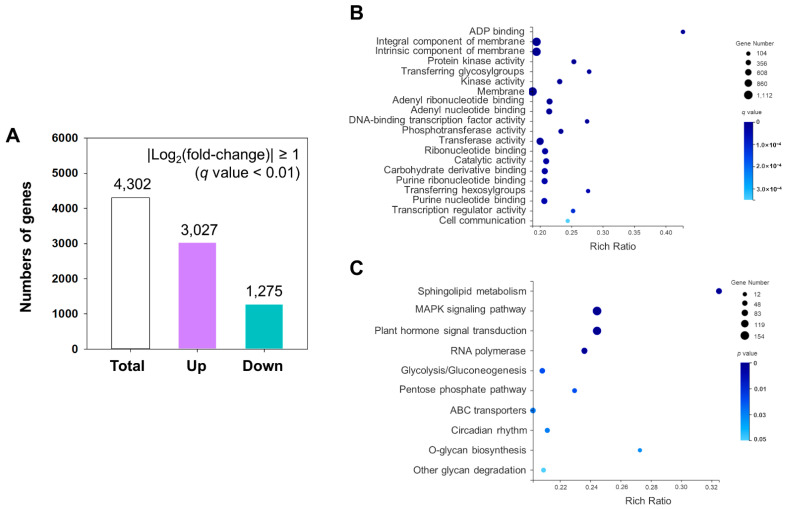
DEGs analysis of *D. catenatum* response to low temperature. (**A**) Overview of upregulated and downregulated genes in *D. catenatum* under cold stress. (**B**) GO enrichment analysis of the DEGs. (**C**) KEGG enrichment analysis of DEGs.

We further determined the differentially expressed genes (DEGs) between *D. catenatum* with and without cold stress using DESeq (*q* value < 0.01); FPKM values were used to represent the gene expression levels. If a gene showed a |Log_2_ (fold-change)| ≥ 1 (*q* value < 0.01) in expression in two contrasting groups, the gene was considered differentially expressed. As shown in Figure 2A, a total of 4302 DEGs were detected in chilled-stress plants compared to control plants. Among these DEGs, the upregulated DEGs were 3208 and the downregulated DEGs were 1275 in cold-treated plants (Table S5).

### 2.3. Analysis of Differential Expressed Genes (DEGs) in Cold Stress

**Figure 3:**
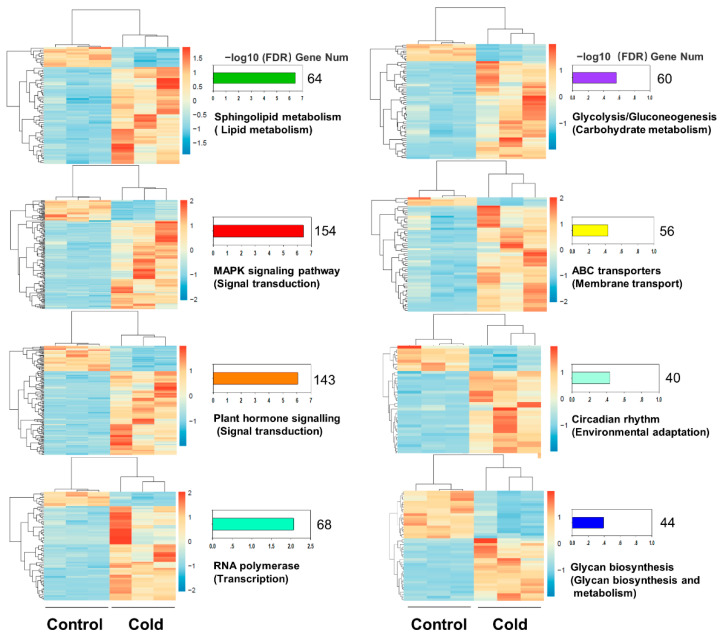
Heatmap of mainly enriched pathways. Bar graph of −log10 transformed FDR values are shown.

The 4302 significantly DEGs were classified into three major categories by GO analysis: 14 molecular functions, 3 cellular components and 17 biological processes (Figure S4). The most enriched GO terms were membrane (GO:0016020), protein kinase activity (GO:0004672), DNA-binding transcription factor activity (GO:0003700), signaling transduction (GO:0007165), regulation of circadian rhythm (GO:0042752), DNA integration (GO:0015074), and response to stimulus (GO:0050896) (Figure 2B; Table S6). KEGG enrichment analysis showed that sphingolipid metabolism (ko00600), MAPK signaling pathway (ko04016), plant hormone signal transduction pathway (ko04075), RNA polymerase (ko03020) and circadian rhythm (ko04712) pathways were most abundantly enriched. Moreover, several polysaccharide metabolic pathways were activated under low temperature, such as glycolysis/gluconeogenesis (ko00010), pentose phosphate pathway (ko00030), and other glycan degradation (ko00511), which indicated that carbohydrate metabolism played a crucial role in responding to cold stress in *D. catenatum* (Figure 2C; Figure S5; Table S7). According to the above analysis, we found that functional enrichments of DEGs responding to cold stress in *D. catenatum* were closely related to several plant key cold tolerance pathways, including protein kinase activity, lipid metabolism, signal transduction, transcription, membrane transport, and environmental adaptation. Meanwhile, we found that carbohydrate metabolism pathways were enriched in *D. catenatum* under cold stress (Figure 3; Table S8).

**Figure 4:**
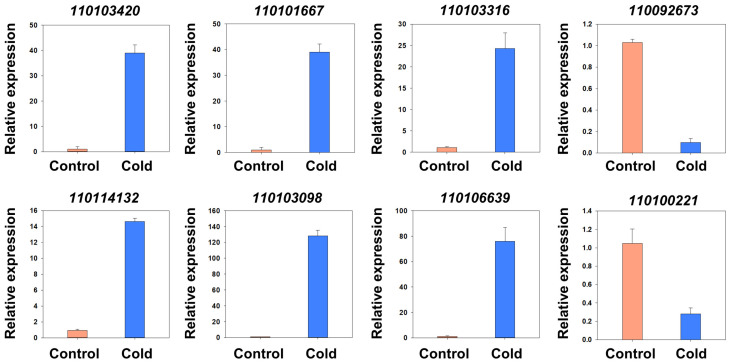
RT–qPCR validation of candidate differentially expressed genes linked to cold resistance in *D. catenatum*. Data are the mean ± SD of three independent biological replicates.

To validate the reliability of the gene expression patterns of DEGs from RNA-Seq data, eight DEGs were randomly selected for analysis by RT–qPCR using gene-specific primers (Figure 4). As expected, the expression levels of these candidate genes obtained by RT–qPCR were consistent with the fold change value of the corresponding genes in RNA-Seq data (Table 1). These results confirm the reliability of the RNA-Seq analysis and reflect the real transcriptomic changes in *D. catenatum* responding to cold stress.

### 2.4. Analysis of Differential AS (DAS) in Response to Cold Stress

**Figure 5:**
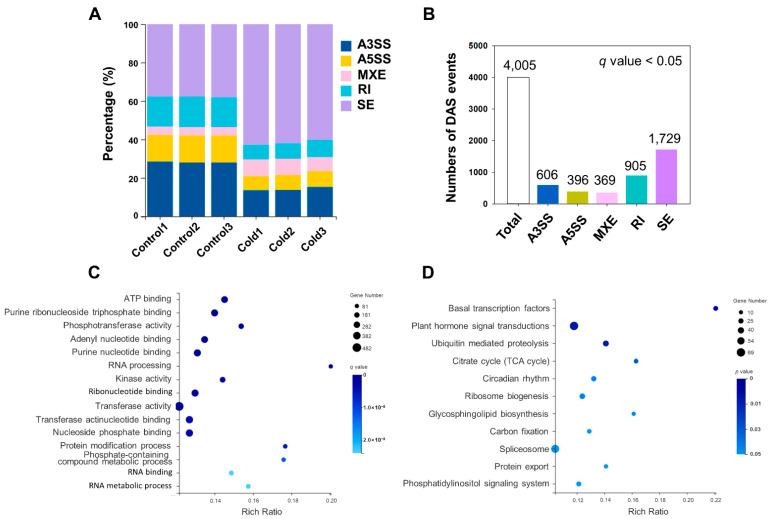
Analysis of differentially alternative splicing events based on RNA-seq data. (**A**) Alternative splicing events predicted in different groups with and without cold stress. (**B**) Numbers of DAS events in *D. catenatum* under cold stress. (**C**) GO enrichment analysis of DAS genes. (**D**) KEGG enrichment analysis of DAS genes.

Among the 22,687 expressed genes, we detected a total of 11,408 and 21,818 alternative splicing events in samples with and without cold treatment, respectively. The RNA-Seq data revealed that the most abundant AS event type in *D. catenatum* under normal and cold conditions was ES (37% and 61%) events. Moreover, the total number of AS events under cold stress was significantly increased compared to those under normal conditions, indicating that cold stress would promote alternative splicing events occurring in *D. catenatum* (Figure 5A; Table S9). Furthermore, we identified a total of 4005 DAS events from 2319 genes in *D. catenatum* under cold stress compared to normal conditions, in which 606 DAS (15%) were A3SS events, 396 DAS (10%) were A5SS events, 369 DAS (9%) were related to MXE events, 905 DAS (23%) were RI events, and the most abundant were ES events, including 1729 DAS (43%) (Figure 5B; Table S10). These results indicate that many genes exhibit two or more AS types and that abundant RI and SE alternative splicing events were susceptible to cold stress.

GO annotation and KEGG pathway enrichment analyses were used to analyse the functional categories of genes with DAS events. According to GO enrichment analysis, the main functional terms of DAS genes were significantly enriched in ATP binding (GO:0005524), purine ribonucleoside triphosphate binding (GO:0035639), RNA binding (GO:0003723), ribonucleotide binding (GO:0032553), RNA processing (GO:0006396), RNA metabolic process (GO:0016070), and mRNA processing (GO:0006397). Protein kinase activity (GO:0004672) and protein modification process (GO:0036211) were also enriched in this process (Figure 5C; Table S11). The KEGG enrichment pathway analysis provided classification of these DAS (Figure 5D; Table S12). DAS from chilled samples were significantly enriched in basal transcription factors (ko03022), plant hormone signal transduction (ko04075), citrate cycles (TCA cycle) (ko00020), circadian rhythm—plant (ko04712), and spliceosome (ko03040). It has been reported that splicing factors can also be alternatively spliced, changing the number of AS isoforms in their downstream targeted genes [34]. Our results indicated that the types of cold-induced alternative splicing events increased in *D. catenatum,* which may be caused by alternative splicing changes in splicing factors under cold stress.

**Figure 6:**
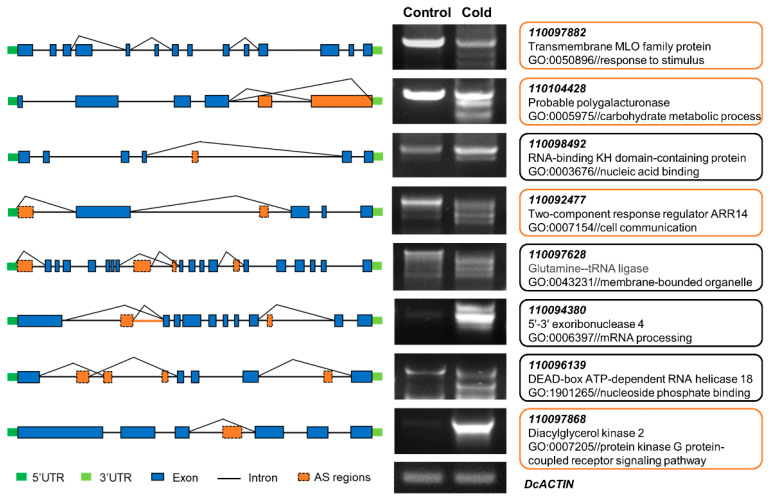
Validation of alternative splicing events in eight selected genes predicted by RNA-Seq through RT–PCR. The left part is gene model diagrams. The medium part is the bands of the products of alternatively spliced genes. *DcACTIN* was used as an internal control. The right part is the annotation of eight selected genes. The red frames indicate the genes with both different expression level and differential alternative splicing events, and the black frames indicate the genes with only DAS events.

To evaluate the reliability of these cold-responsive splicing events, RT–PCR was performed on eight selected genes (predicted with IR/ES events) using RNA isolated from *D. catenatum* leaves (with and without cold stress) (Figure 6). The results showed that cold stress induced more AS events or changed the isoforms of AS events compared to normal plants. These differences resulted from exon or skipping intron retention by sequencing and BLAST. The alternative splicing patterns of these eight examined genes by RT–PCR were highly consistent with RNA-Seq, which confirms the reliability of our RNA-Seq analysis and indicates that AS events were promoted in *D. catenatum* under cold conditions.

### 2.5. Comparative Analysis of DEGs and DASGs in Response to Cold Stress

**Figure 7:**
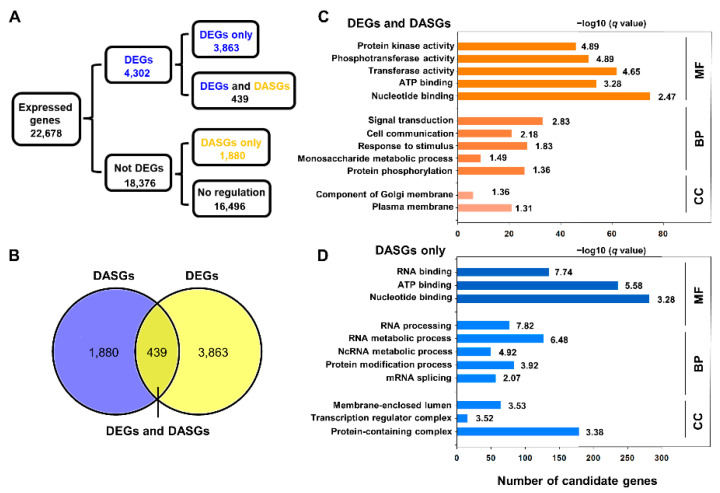
Analysis of differentially expressed (DE) and differential alternative splicing (DAS) genes in cold stress. (**A**) Flow chart showing the statistics analysis of the DE and DAS genes. (**B**) Venn diagrams showing the overlap of genes with DAS events and DE genes. (**C**) GO enrichment of genes with both DE and DAS in response to cold. (**D**) GO enrichment of genes with differential alternative splicing only in response to cold. Bar graph of −log10 transformed *q* values are shown. MF, molecular function; BP, biological process; CC, cellular component.

We further compared the genes that exhibited differential expression levels and different alternative splicing events. We found a total of 439 genes (~19%) with both DAS events and different expression levels and 1180 genes (~81%) that were only regulated by AS (not DE) (Figure 7A,B; Table S13). In addition, ~10% of the 4302 DEGs were differentially alternatively spliced. The DE and DAS gene groups were significantly different, with an overlap of only 439 genes. To explore the effect of cold-responsive alternative splicing on biological processes, we analysed the function of 439 genes with both DE and DAS events in response to cold. The GO enrichment analysis revealed that these genes were mainly related to the typical cold tolerance pathway (Figure 7C). The most significant enrichment terms were protein kinase activity (GO:0004672), signal transduction (GO:0007165), cellular response to stimulus (GO:0051716) and plasma membrane (GO:0005886). We identified a transmembrane MLO family protein (*110097882*) involved in the response to stimulus. The expression level of this gene was significantly downregulated and generated more alternative splicing isoforms under cold stress by RT–PCR (Figure 6), indicating that the AS gene may affect its transcript abundance and alter its AS isoforms to decelerate the stress response to cold. In contrast, according to GO enrichment annotation, genes only with DAS events under cold stress were involved in RNA processing (GO:0006396), RNA binding process (GO:0003723), RNA metabolic process (GO:0016070) and protein modification process (GO:0036211) (Figure 7D). For instance, we isolated a 5′-3′ exoribonuclease 4 (*110094380*) and detected its AS events by RT–PCR, demonstrating that it is involved in mRNA processing. As shown in Figure 6, this gene generated more than one type of AS event, and the transcript level of AS isoforms was significantly upregulated, suggesting the involvement of splicing-related genes during cold responses. These results strongly indicate that genes regulating AS events, such as spliceosome and splicing factors, could affect the alternative splicing of downstream genes by being alternatively spliced under cold stress. We proposed that some genes with both DAS and DE events, such as genes involved in the protein kinase process, can be differentially regulated to influence their functions in cold stress. Thus, we concluded that the quantity of alternative splicing events in some cold-specific genes can change the expression patterns of corresponding genes and finally affect the cold tolerance response.

### 2.6. DcCBP20 of D. Catenatum Modulates the Alternative Splicing of Genes Responding to Cold Stress

**Figure 8:**
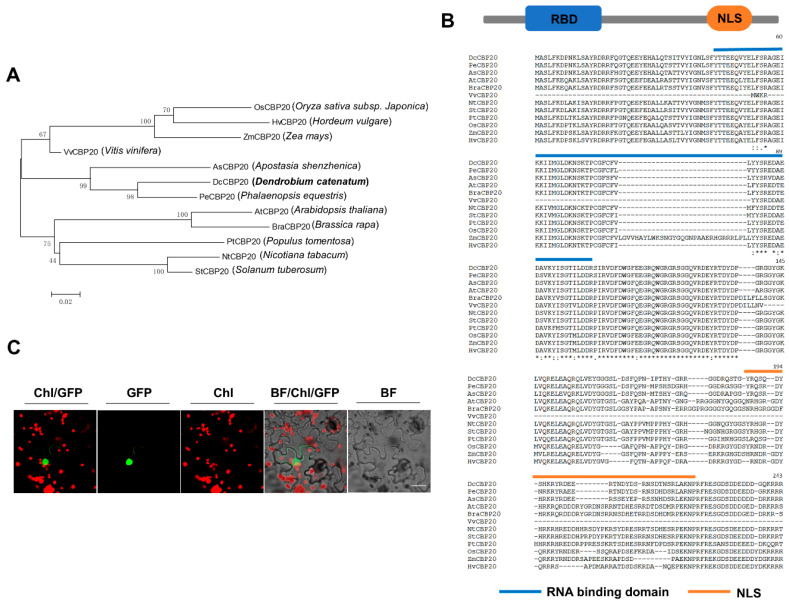
Functional analysis of *CBP20* homologues in *D. catenatum*. (**A**) Phylogenetic analysis of *CBP20* homologues in six representative dicotyledonous species and five monocotyledonous species. (**B**) Sequencing alignment and conserved motif prediction of *CBP20* homologues. (**C**) Subcellular localization of DcCBP20-GFP in tobacco leaves. The *DcCBP20-GFP* construct was transiently expressed in the leaves of *Nicotiana benthamiana* (bar = 10 μm), and GFP fluorescence was observed.

A previous study revealed that the proportion of uncapped transcripts that were alternatively spliced was significantly increased when exposed to cold stress [35]. *CBP20*, as an important subunit of the nuclear cap-binding complex (CBC), can improve the stability of transcripts [25]. Meanwhile, it has been reported that *CBP20* is involved in drought and salt stress by regulating alternative splicing events [29]. In this study, we found that *Arabidopsis cbp20* mutant plants showed great sensitivity to chilling stress at 4 °C (Figure S6), which indicated that *CBP20* may be required for cold tolerance in plants. Thus, using the Download BLAST Software and Databases of NCBI (https://ftp.ncbi.nih.gov/blast/executables/igblast/release/LATEST/, accessed on 1 January 2022), the full-length CDS of *AtCBP20* (*AT5G44200*) was as a “Query” sequence to extract the homologous *DcCBP20* from the *D. catenatum* CDS database. We identified and cloned the homologous gene of *CBP20* in *D. catenatum*, which was only a single gene. Sequence alignment and phylogenetic analysis found that *CBP20* showed high similarities with the *CBP20* sequences of two *Orchidaceas* species, *Apostasia shenzhenica* and *Phalaenopsis equestris* (Figure 8A). We further found that *DcCBP20* exhibited two conserved motifs: an RNA binding domain (RBD) motif and a NLS motif (Figure 8B). We subsequently generated the *35S::DcCBP20-GFP* construct and detected the subcellular localization of the DcCBP20 protein using a transient expression assay in *N. benthamiana*. As shown in Figure 8C, GFP fluorescence was detected only in the nucleus, which is consistent with the subcellular location of *CBP20* in *Arabidopsis*.

**Figure 9:**
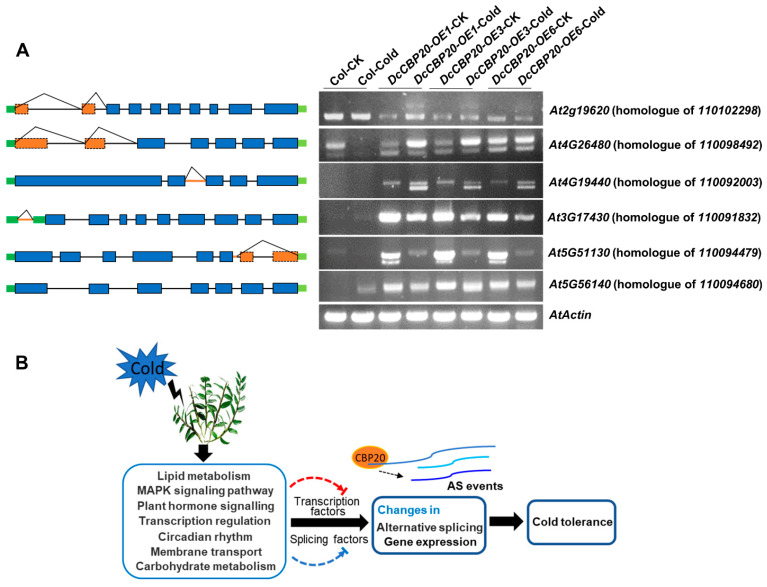
Function of *CBP20* homologous gene of *D. catenatum* in response to cold. (**A**) The splicing isoforms of the PCR products using specific primers in cDNA of *DcCBP20* transgenic *Arabidopsis* detected by RT–PCR. The wild type and *DcCBP20* transgenic *Arabidopsis* were treated with or without 4 °C for 12 h, three independent transgenic lines of *DcCBP20-OE* were used for RT-PCR analysis. (**B**) A model of the cold signaling pathway in *D. catenatum*. Under cold stress, differentially expressed genes responding to cold stress in *D. catenatum* by RNA-Seq were mainly related to lipid metabolism, protein kinase activity, plant hormone signaling transduction, transcription regulation, circadian rhythm, membrane transport and carbohydrate metabolism. These processes activate or suppress transcription factors and splicing factors, which respond to cold stress. These factors further function in the alternative splicing and gene expression of downstream targets. During this process, *CBP20* mediates alternative splicing events to respond to cold stress in *D. catenatum*.

To further investigate whether *DcCBP20* influences AS events under cold stress, we generated transgenic lines overexpressing *DcCBP20* in Col-0 *Arabidopsis* (Figure S7). The phenotype of the transgenic plants expressing the *DcCBP20* was similar to the wild type Col-0 plants, and the DcCBP20-GFP protein was detected in independent transgenic lines using GFP antibody. Then, chilling tolerance tests among *Arabidopsis* wild-type Col-0 and three independent *DcCBP20-OE* transgenic lines were performed at 4 °C for 12 h. We subsequently blasted the homologues of some candidate genes associated with the cold tolerance response of *D. catenatum* in *Arabidopsis*, including three DAS only and three DE ± DAS genes identified and the homologous genes in validation the reliability of RNA-Seq as previously described. Then, we compared the AS variants of these candidate genes in Col and three independent *DcCBP20-OE* plants by RT–PCR (Figure 9A). We found that several genes showed significantly different AS isoforms in *DcCBP20-OE* transgenic plants in comparison with wild-type plants. Moreover, the abundance and variants of AS in *DcCBP20* transgenic plants were altered after cold treatment, and the AS isoforms changes were much similar among three independent transgenic lines. These genes include the gene encoding a pentatricopeptide repeat-containing protein (*At4G19440*, homologous gene of *110098492* in *D. catenatum*), which is involved in the transferase activity pathway; we found that the expression level of this gene increased in *DcCBP20-OE* plants compared to Col-0 *Arabidopsis* under control condition, and a newly shorter AS product with higher expression level was generated in *DcCBP20-OE* plants after cold stress compared to Col-0 *Arabidopsis*. Furthermore, we found a gene encoding a nucleotide/sugar transporter family protein (*At3G17430*, homologous gene of *110091832* in *D. catenatum*) that showed significant differences between Col-0 and *CBP20-OE* transgenic plants. The transcript products nearly can’t be detected in Col-0 with and without cold stress, new transcript products with high expression level were generated in *DcCBP20-OE* transgenic plants with and without cold stress, which indicated that *DcCBP20* contributed to resist cold stress through enhancing the expression level of protein in the sugar transport pathway. AS differences of the gene (*At5G51130*, homologous gene *110094479* of in *D. catenatum*) were also detected. The abundance of the transcript product of this gene was dominantly increased, and a newly short AS variant was generated in *DcCBP20-OE* plants compared to Col-0 *Arabidopsis*. This gene encoded an S-adenosyl-L-methionine-dependent methyltransferases superfamily protein, indicating that *DcCBP20* contributed to resist cold stress through activating the protein involved in transferase activity. We also detected the alternative splicing changes of homologous genes of seven genes used to validate the reality of RNA-Seq. Among the seven genes described previously, only one gene (*At4G26480*, homologous gene of *110098492* in *D. catenatum*) showed significantly AS variants differences among Col-0 and *DcCBP20-OE* transgenic plants; this gene belonged to the RNA binding pathway. We found that there was a new isoform of AS variant generated in *DcCBP20-OE* plants after cold; the expression level of two alternative splicing isoforms was significantly improved in cold stress, which was similar to AS variants changes in *D. catenatum,* indicating that this gene involved in a similar alternative splicing progress in *Arabidopsis* and *D. catenatum*. The other six genes didn’t show significant differences in AS isoforms or expression level between Col-0 and *DcCBP20-OE* plants, or was not detected (Figure S8). Considering the differences of species, homologues and transgenic plants, these results are reasonable.

Above all, these observations of significantly different AS isoforms in the six genes between *DcCBP20-OE* transgenic plants and wild-type plants strongly suggested that *DcCBP20* was involved in alternative splicing events in cold stress. Furthermore, *DcCBP20* played important roles in chilling response in *D. catenatum* by altering AS isoforms and enhancing the abundance of alternative splicing of some specific cold-specific genes for cold tolerance.

## 3. Discussion

*Dendrobium catenatum Lindl* is a valuable herbal medicine that is popular due to its special efficacy and medicinal value. Most *D. catenatum* cultivars have adapted to warm southern climates; the ability to tolerate cold is a major bottleneck restricting their cultivation [31]. Understanding the molecular mechanism of cold tolerance and breeding cold-tolerant cultivars is required for *D. catenatum* to adapt to lower temperatures when expanding cultivated regions to northern areas. In the present study, we analysed the cold stress response of *D. catenatum* and found that AS played an important role in the cold tolerance response.

Alternative splicing is an important posttranscriptional process that increases the diversity of proteins and impacts mRNA stability. Alternative splicing events are highly modulated to adapt to environmental stress in plants. AS has been related to cold stress resistance in plants, and previous studies reported that alternative splicing events were largely regulated by heat and drought stress [21,22,36]. In our study, we performed RNA-Seq to analyse the gene expression differences and alternative splicing variants in *D. catenatum* responding to cold stress. A total of 4302 genes with different expression level under cold stress were detected, and these DEGs were related to protein kinase activity, membrane transport, plant hormone signaling, transcription, and circadian pathways, which are consistent with the important cold-tolerant pathways in plants. These results indicate that cold stress causes membrane lipid changes and protein modification changes to improve cold tolerance; plant hormones may play a major role in *D. catenatum* resistance to cold stress. Furthermore, we found that glycan biosynthesis and metabolism pathways were significantly enriched in *D. catenatum* under cold stress, indicating that polysaccharides rich in leaves and stems of *D. catenatum* are involved in the process of cold resistance. These results suggested that *D. catenatum* exhibits special cold-resistant pathways related to polysaccharides.

For differential alternative splicing events, we found that exon skipping was the most abundant splicing event in the *D. catenatum* response to cold stress; intron retention was also strongly induced. Previous studies have clearly proven that ES splicing events promote the generation of protein variants [19]. A total of 2319 DAS genes were identified and mainly related to RNA processing, RNA binding and spliceosomes. We further compared those DEGs and DASGs and found a total of 439 genes with both differential alternative splicing events and different expression patterns in cold stress, indicating that these differential alternative splicing products could alter the abundance of functional transcripts to change the functions of protein-coding genes under cold stress. A total of 1180 genes were only differentially spliced, and these genes were mainly splicing factors involved in mRNA metabolic and splicing processes. We determined that some splicing factors, such as genes encoding mRNA processing and nucleoside phosphate binding, generated several new AS isoforms and were differentially regulated. These results indicate that cold-induced changes in the types of transcript products of splicing factors would alter the expression levels of splicing factors themselves and finally affect the transcription and alternative splicing events of downstream targeted genes in the cold stress response in *D. catenatum*.

A recent study revealed that most of the cold-induced DAS events contained PTCs (premature termination codons), and half of the transcripts involved in DAS events were degraded to decrease the expression level of transcripts during the repression of cellular processes. The degradation proportion of cold-induced genes could be increased due to mRNA alternative splicing imbalance, which can decrease the cold stress tolerance of casava [35]. The CBP complex, including *CBP20* and *CBP80*, plays a key role in posttranscriptional processes, including splicing, transcription and translation [25]. The stability of mRNA can be regulated by cap-binding proteins, such as the CBC complex, by competing with decapping enzymes. This competition could mediate the balance between transcription elongation and transcripts degradation [25,37]. Moreover, *CBP20* has been reported to be involved in alternative splicing events in plants [31]. In this study, we found that overexpression of *CBP20* in *D. catenatum* in *Arabidopsis* plants significantly influenced AS events under cold stress. *DcCBP20* contributed to the generation of more alternative splicing isoforms of cold-induced genes compared to wild-type *Arabidopsis* and enhanced the abundance of original alternative splicing isoforms. These genes are abundantly involved in the internal membrane component pathway, RNA binding pathway, and transferase activity pathway. These new alternative splicing isoforms regulated by *DcCBP20* in cold stress may play roles in the response of *D. catenatum* to cold stress.

## 4. Materials and Methods

### 4.1. Plant Materials and Cold Treatments

The *D. catenatum* cultivar used in this study was harvested from Xishuangbanna Tropical Botanical Garden. Tissue culture seedlings (2 months) in tissue culture bottles were separated from the medium and transplanted into pots filled with growth matrix (pine bark). The plants were kept in a greenhouse at 25 °C under long-day (LD) conditions (16–8 h light/8-h dark). The cold stress tests of *D. catenatum* were performed in a climate chamber at 4 °C or −4 °C under the same photoperiod. The control plant samples were grown under 25 °C, and the other conditions remained same. For cold stress experiment, we performed three independent control or treatment experiments. For each treatment (or control) experiment, three biological replicates of three plants of per sample type for each assay were performed. *Arabidopsis* wild-type Col-0, mutant and transgenic seeds were grown on Murashige and Skoog (MS) plates at 25 °C under a 16-h light/8-h dark photoperiod condition and then transferred into the greenhouse.

### 4.2. Measurements of Relative Electrolyte Leakage and Chl Fluorescence

Relative electrolyte leakage, as an important indicator of membrane permeability, was measured by the method according to Jiang [38]. Briefly, 100–200 mg of leaves from *D. catenatum* with and without cold treatment were rinsed with ddH_2_O, then were placed into clean test tubes with 10 mL ddH_2_O. The above tubes were put into a vacuum pump for 30 min, and then incubated at room temperature for 1 h with shaking and mixing. Using a conductivity meter, the electrical conductivity of the solution (C1) was measured. Then, the tubes were boiled for 10 min, the electrical conductivity (C2) was measured again after being cooled to room temperature. According to the formulas REL = C1/C2 × 100%, Injury degree= (REL_t_ − REL_ck_)/(1 − REL_ck_) × 100%, the electrical leakage was calculated by injury degree. All measurements were repeated in three independent experiments. Data are expressed as the means ± standard deviation (SD) of three biological replicates. Chl fluorescence was determined through an IMAGING-PAM Chl fluorimeter, and the *Fv*/*Fm* ratios were measured by Imaging WinGigE software (MAXI Version, Walz, Germany), based on three plants for each sample type. The plants were dark-adapted for 30 min. Student’s *t* test was used for comparison of difference between control and treatment. **, *p* < 0.01; and *, *p* < 0.05.

### 4.3. RNA Isolation, Sequencing and Transcriptome

Total RNA was extracted from the leaves of *D. catenatum* with and without chilling stress using the CTAB-LiCl method according to the GASIC method with some improvements [39]. RNA purity was detected using a Nanodrop Spectrophotometer 2000, and RNA degradation was detected by 1% agarose electrophoresis. For both the control and the chilling treatment sample, three biological replicates were harvested at the same time. For each replicate, the leaves from three plants were harvested. After testing the quality of RNA samples, an RNA-seq library was constructed and sequenced using a MGISEQ-2000 sequencing instrument by BGI Co. (Shenzhen, China). The raw reads obtained by Illumina sequencing were filtered using SOAPnuke software (v1.4.0, -l 15 -q 0.2 -n 0.1), the reads with adaptors were discarded, and low-quality reads containing more than 5% ambiguous “N” bases and *q* value < 20 were discarded, after which the remaining reads were considered as clean reads [40]. Then, the clean reads were mapped to the reference genome (http://orchidbase.itps.ncku.edu.tw/est/Dendrobium_2019.aspx, accessed on 5 June 2021) by HISAT (Hierarchical Indexing for Spliced Alignment of Transcripts) software (http://www.ccb.jhu.edu/software/hisat, accessed on 1 January 2022) [41].

### 4.4. Analysis of Functional Enrichment of DEGs

The FPKM (fragments per kilobase of exon per million mapped fragments) method was used to detect the expression level of each transcript. Bowtie2 software (http://bowtie-bio.sourceforge.net/Bowtie2/index.shtml, accessed on 1 January 2022) was applied to map clean reads to reference gene sequences, and then RSEM (http://deweylab.biostat.wisc.edu/rsem/rsem-calculate-expression.html, accessed on 1 January 2022) was used to calculate the gene expression level of each sample to obtain the FPKM values [42,43]. An FDR rate *q* ≤ 0.01 and |Log2 (fold-change)| ≥ 1 were considered as criteria to identify differentially expressed genes (DEGs). DEG analysis was performed using the DESeq2 method [44].

GO annotation was carried out using Gene Ontology software (GO; http://geneontology.org, accessed on 1 January 2022) [45]. GO enrichment annotation of DEGs was analysed with the phyper package in R software, and a *q* value ≤ 0.05 was the criterion for significant enrichment. KEGG pathway functional enrichment of DEGs was implemented using the same method (https://www.genome.jp/kegg/, accessed on 1 January 2022) [46], and a *p* value ≤ 0.05 was the criterion for significant enrichment.

### 4.5. Analysis of Functional Enrichment of DAS

rMATS (http://rnaseq-mats.sourceforge.net, accessed on 1 January 2022) was used to identify five basic types of AS events, SE, MXE, A5SS, A3SS and RI, from RNA-Seq clean reads [47]. DAS events were identified between samples with and without chilling stress using rMATS software. An FDR *q* value ≤ 0.05 was set as the criterion for DAS.

### 4.6. RT–qPCR and RT–PCR

The quantitative real-time PCR experiments were performed as reported previously [48]. Total RNA was isolated from the leaves of *D. catenatum* with and without chilling stress using an improved CTAB-LiCl method. For each sample type, we harvested the leaves for three biological replicates. For each replicate, the leaves from three plants were harvested. First strand cDNA was subsequently synthesized from 1.5 μg of DNase-treated RNA in a 20 μL reaction volume using a GoScript reverse transcription system kit (Promega). Then, RT–qPCR was conducted using Fast Start Universal SYBR Green Master Mix (ROX) on an Applied Biosystems 7500 machine according to the manufacturer’s instructions. At least three biological and separately three technical replicates for each cDNA sample were used in the RT–qPCR analysis. The data are expressed as the mean ± SD of the three values of three biological replicates. *DcACTIN* was used as an internal control. All gene-specific primers used in RT-qPCR are listed in Supplemental Table S14.

RT–PCR was applied to candidate genes to validate the AS isoforms. Then, RT-PCR products were detected using 1.5% agarose gels. The gene-specific primers used for amplification of AS variants are described in Supplemental Table S14.

### 4.7. Generation of Transgenic Lines

The full-length cDNA coding region of the *DcCBP20* gene was amplified and then cloned into the *PRI101-GFP* vector between the SailI and EcoRI sites using the In-Fusion cloning system (Clontech), termed *35S::DcCBP20-GFP*. The construct was transformed into *Agrobacterium tumefaciens EHA105*; wild *Arabidopsis* Col was transformed using the *Agrobacterium*-mediated floral-dipping method to generate the corresponding transgenic line. The analysis was subsequently performed with T2 transgenic plants. The primers used are listed in Supplemental Table S14.

### 4.8. Protein Immunoblotting

Protein was isolated from leaves of transgenic *Arabidopsis* in a protein extraction buffer (100 mM Tris-HCl, 20% glycerol, 4% sodium dodecyl sulfate, 0.2% bromophenol blue, 200 mM DTT) and then boiled for 10 min and centrifuged at 12,000 g at 4 °C for 1 min. Total protein with the same volume was loaded onto SDS–PAGE gels, transferred onto PVDF blotting membranes, and then probed with the appropriate primary anti-GFP antibody (1:3000, Clontech) and horseradish peroxidase-conjugated goat anti-mouse secondary antibody (1:3000, Promega). The signal was detected using an imaging device (Tanon 5200).

### 4.9. Transient Expression Assays

A transient transformation assay was applied using previous methods [49]. Briefly, the construct *35S::DcCBP20-GFP* was transformed into *Agrobacterium tumefaciens GV3101*. A. tumefaciens containing *35S::DcCBP20-GFP* was infiltrated into *Nicotiana benthamiana* leaves. After 2–4 days of transformation, a confocal laser scanning microscope (Olympius) was used to detect the fluorescence signal of the GFP fusion protein.

### 4.10. Phylogenetic Analysis and Conserved Motifs Prediction of CBP20

The sequences of *CBP20* used in this study were blasted from Plant Genome (https://www.plabipd.de/plant_genomes_pa.ep, accessed on 1 January 2022) and UniProtKB (https://www.uniprot.org/, accessed on 1 January 2022). The phylogenetic tree was constructed with amino acid sequences using MEGA 7.0 software to implement the NJ (neighbour-joining) method with a bootstrapping value of 1000. The conserved motifs of the CBP20 protein were analysed using Pfam35.0 (http://pfam.xfam.org/, accessed on 1 January 2022).

## 5. Conclusions

In the present study, we found that differentially expressed genes responding to cold stress in *D. catenatum*, as determined by RNA-Seq, were mainly related to lipid metabolism, protein kinase activity, plant hormone signaling transduction, transcription regulation, circadian rhythm, membrane transport and carbohydrate metabolism. We also explored the differential alternative splicing events in *D. catenatum*. We found that the number of AS events was massively increased in cold stress, and all five kinds of AS classes existed in *D. catenatum*. The most enriched type was ES events, which increased the number of transcripts and protein variants. These results indicate that AS events can fine-tune the expression levels and abundance of alternative spliced transcripts responding to cold stress. We further identified and cloned *CBP20* homologues in *D. catenatum* and first found that *DcCBP20* altered the variants and abundance of alternative splicing isoforms of some cold-induced genes in response to cold stress (Figure 9B). Further investigation of how AS influences cold resistance in *D. catenatum* and the correlation of TFs and SFs in regulating the stress response will be valuable to explore the cold stress response of plants.

## Figures and Tables

**Table 1:** Candidate differentially expressed genes in *D. catenatum* transcriptome.

Gene ID	Gene Annotation	Log2 (Cold/ck)	*q* Value (Cold-vs-ck)
*110103420*	Protein TIFY 5A	5.882954842	1.15 × 10^−16^
*110101667*	Two-component response regulator-like APRR9	5.75307647	1.14 × 10^−148^
*110103316*	WRKY transcription factor 50	5.270290384	7.89 × 10^−17^
*110092673*	Cyclin-P4-1-like	−3.506757892	2.00 × 10^−3^
*110114132*	WRKY transcription factor 48	5.583790123	3.73 × 10^−47^
*110103098*	Gibberellin 2-β-dioxygenase 1	6.673964409	2.76 × 10^−60^
*110106639*	polygalacturonase inhibitor 2-like	8.403207013	3.38 × 10^−117^
*110100221*	Alpha-humulene synthase-like	−3.539482498	2.38 × 10^−6^

## Data Availability

All data generated in this study were included in the main article and its supplementary files. All the transcriptome data have been deposited in the NCBI’s BioProject with accession No. PRJNA783177 (Available online: https://www.ncbi.nlm.nih.gov/bioproject/PRJNA783177/, accessed on 1 January 2022).

## Supplementary Materials

The following are available online at https://www.mdpi.com/article/10.3390/ijms23020981/s1.
