# Supplementary material for: Cold Response Transcriptome Analysis of the Alternative Splicing Events Induced by the Cold Stress in D. catenatum
Source: Int J Mol Sci. 2022 Jan 17;23(2):981. doi: 10.3390/ijms23020981 (PMC8778272; doi:10.3390/ijms23020981)
Supplement: Supplementary file 1 [file ijms-23-00981-s001.zip › ijms-1515499-supplementary Figures.pdf]

International Journal of
Molecular Sciences

MDPI

Article

Cold Response Transcriptome Analysis of the Alternative Splicing Events Induced by the Cold Stress in *D. catenatum*

Yan Zheng [1,†], Landi Luo [2,†], Qian Chen [1], Danni Yang [1,3], Yuqiang Gong [1,4], Ya Yang [1], Xiangshi Qin [1], Yuhua Wang [1], Xiangxiang Kong [1,*] and Yongping Yang [1,2,*]

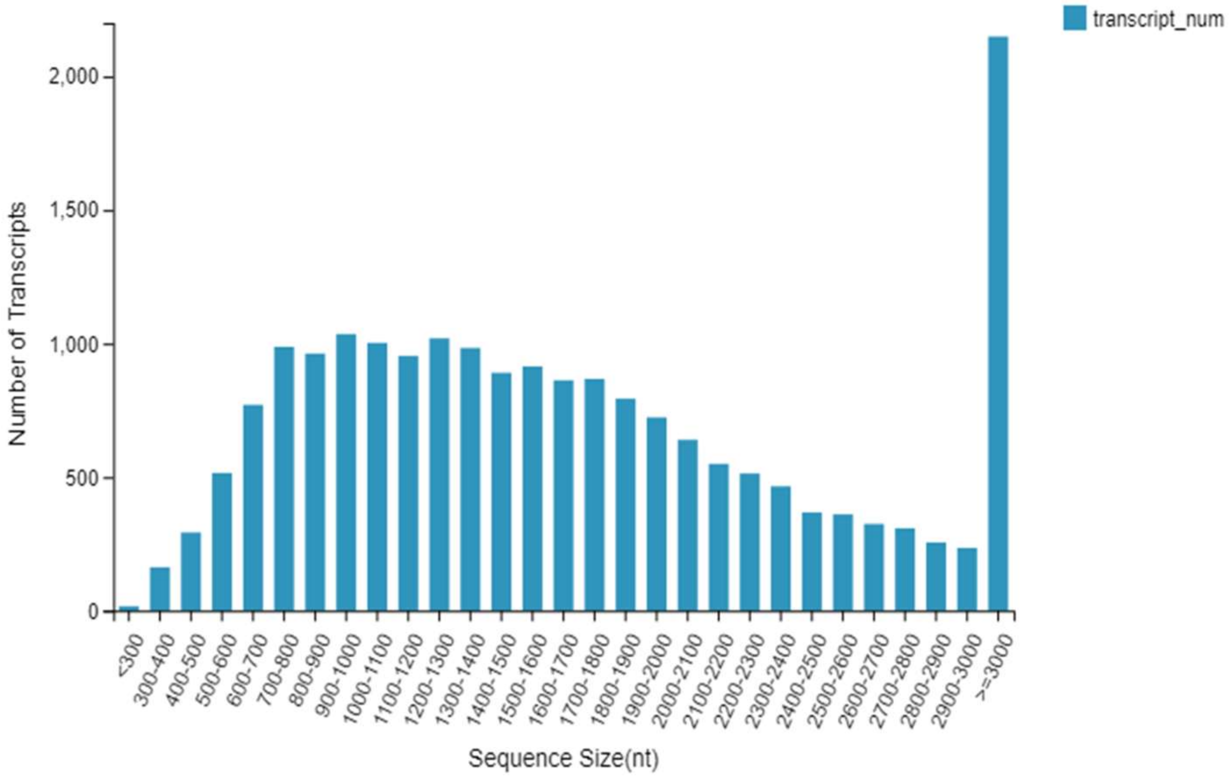

Figure S1. Distribution of transcripts lengths in *D. catenatum* transcriptome.

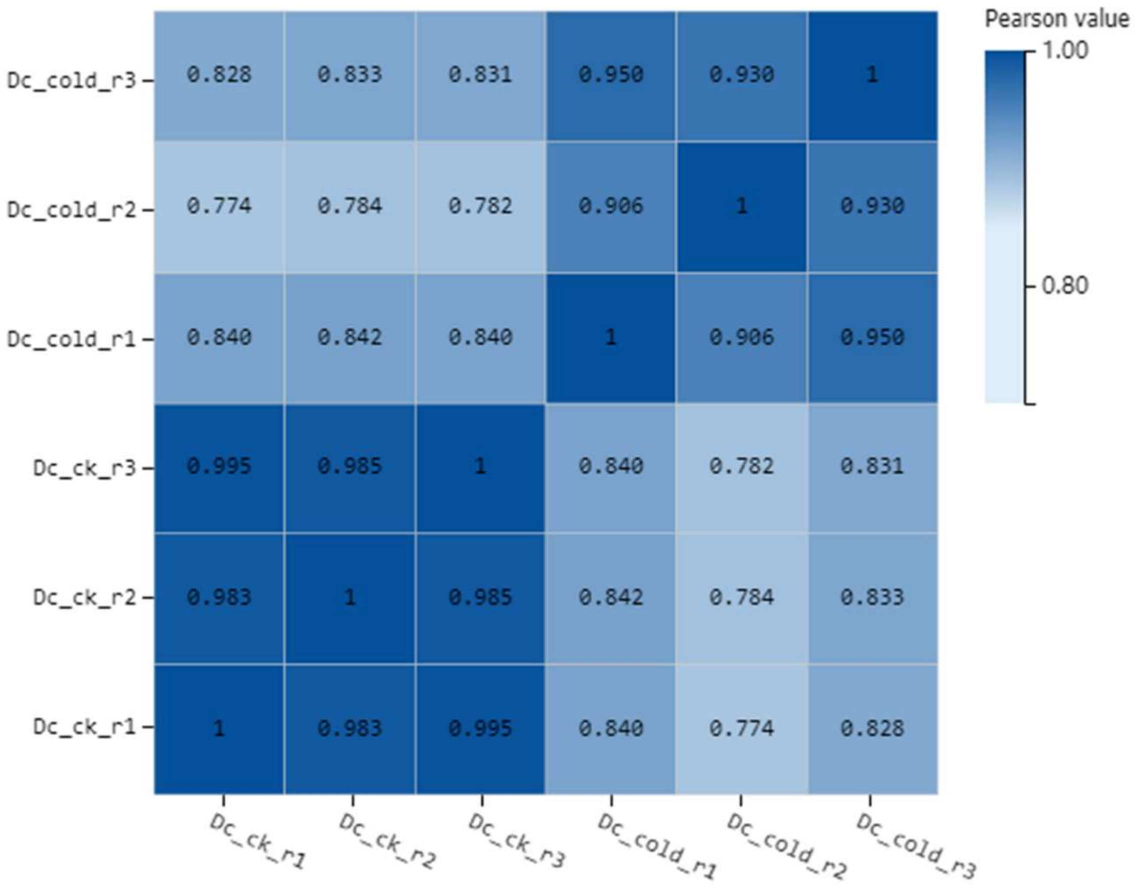

Figure S2. Heatmap of Pearson correlation analysis among different samples.

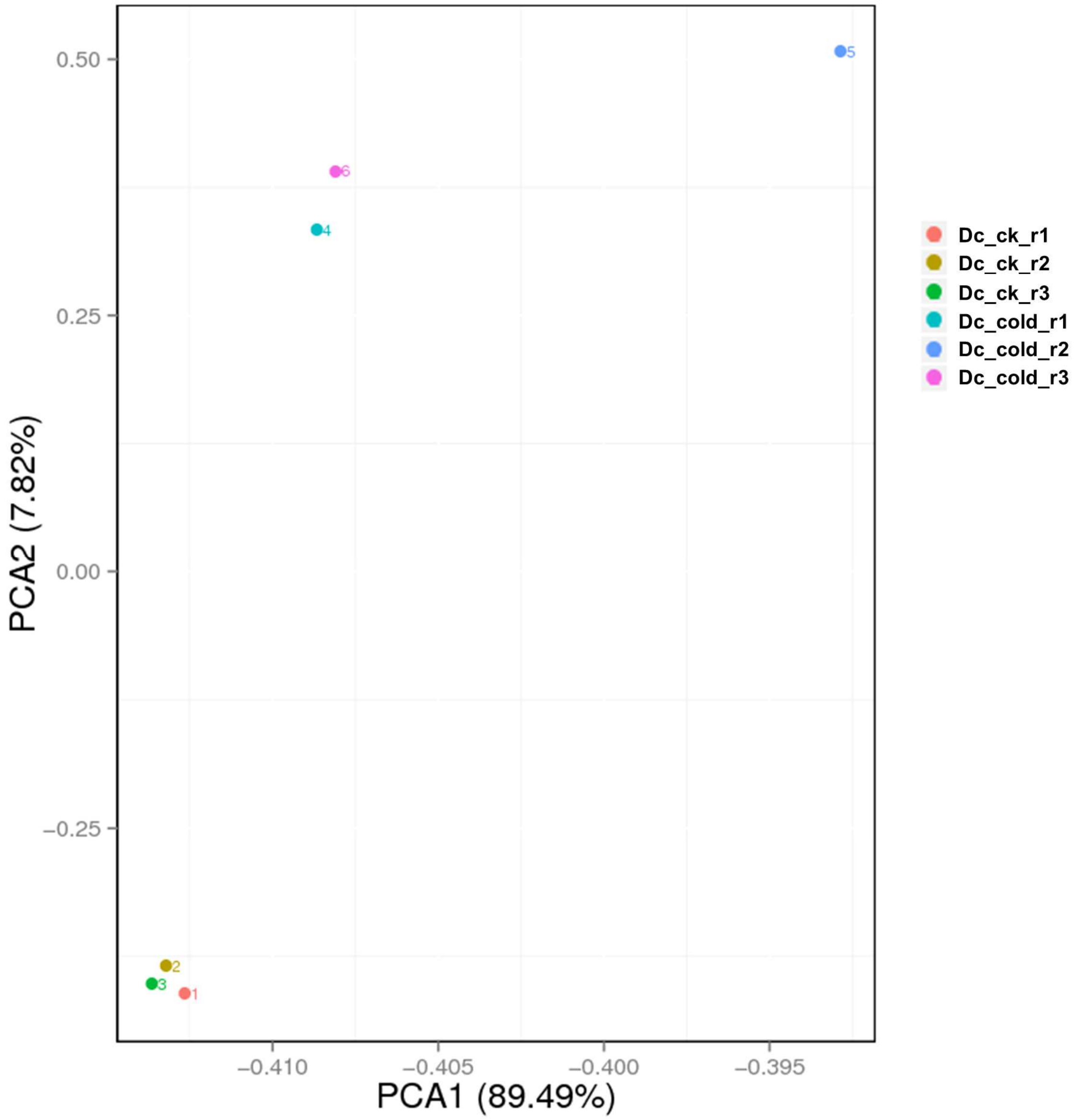

Figure S3. Principal component analysis (PCA) factorial maps of transcriptome data.

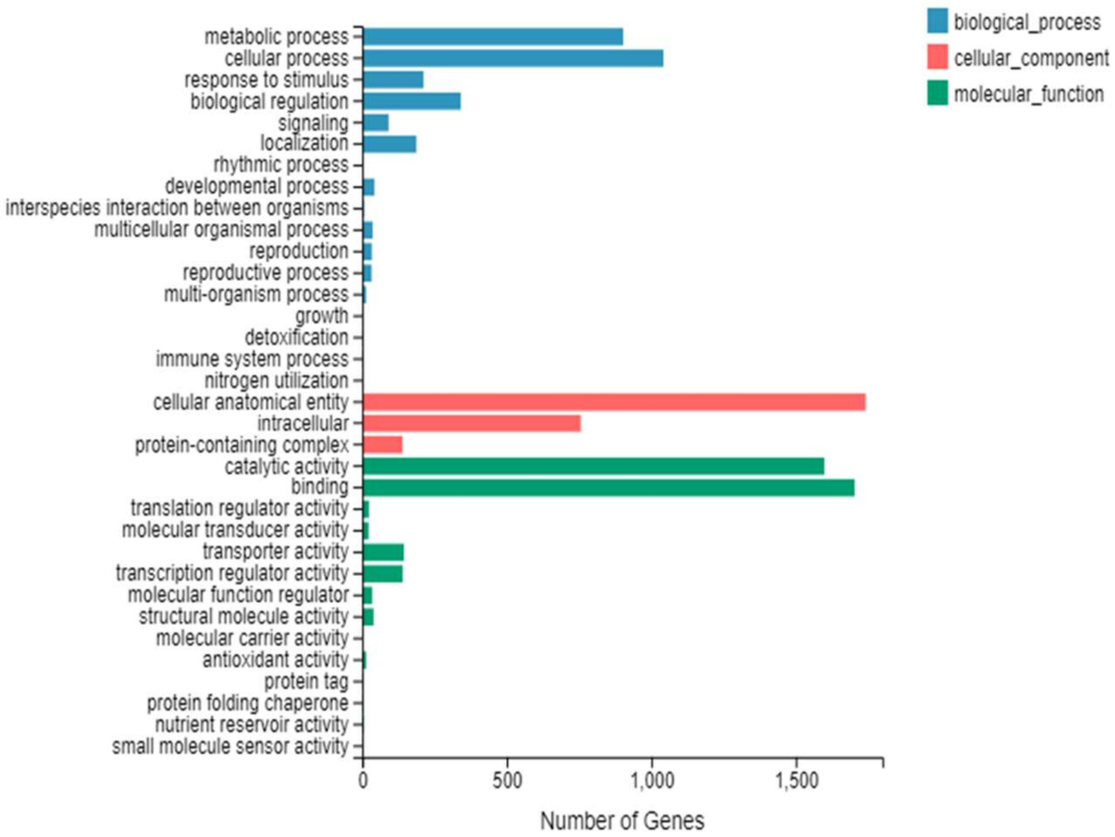

Figure S4. GO annotation of differentially expressed genes in *D. catenatum* in response to cold.

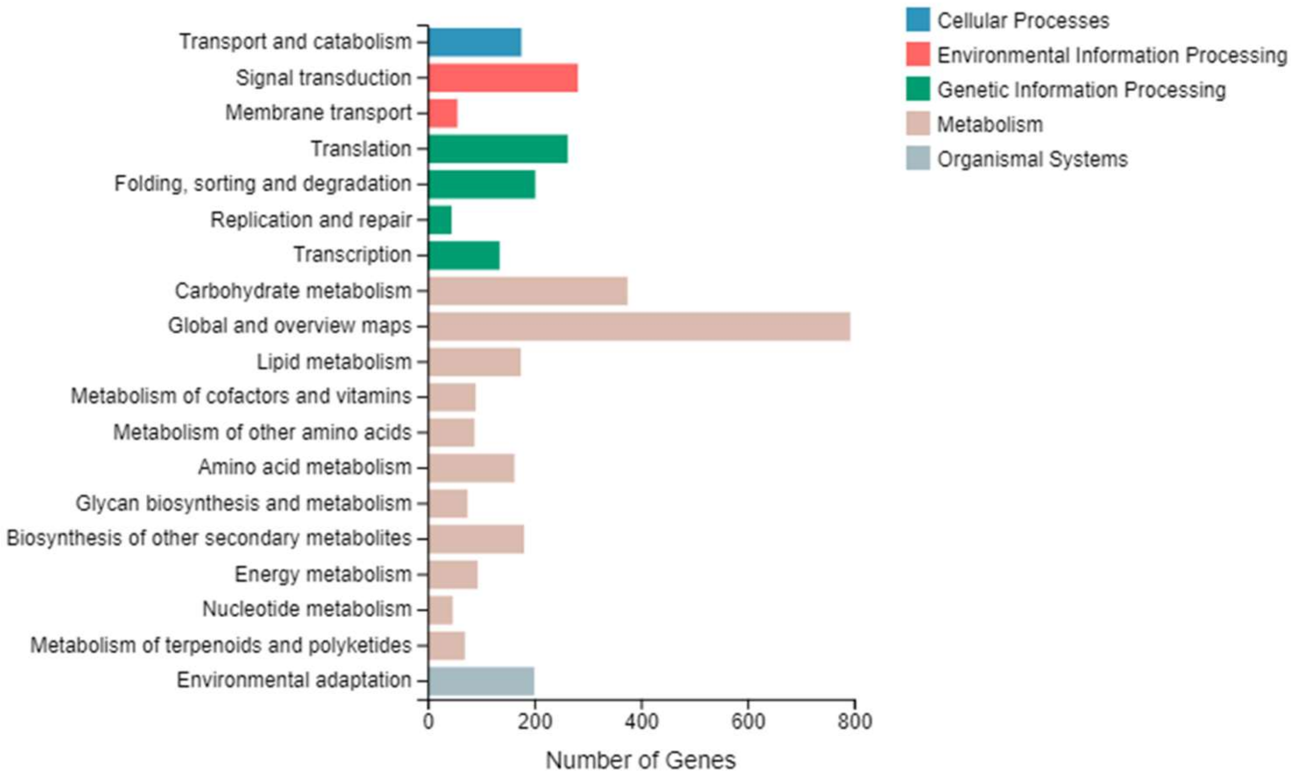

Figure S5. KEGG pathway annotation for differentially expressed genes in *D. catenatum* in response to cold.

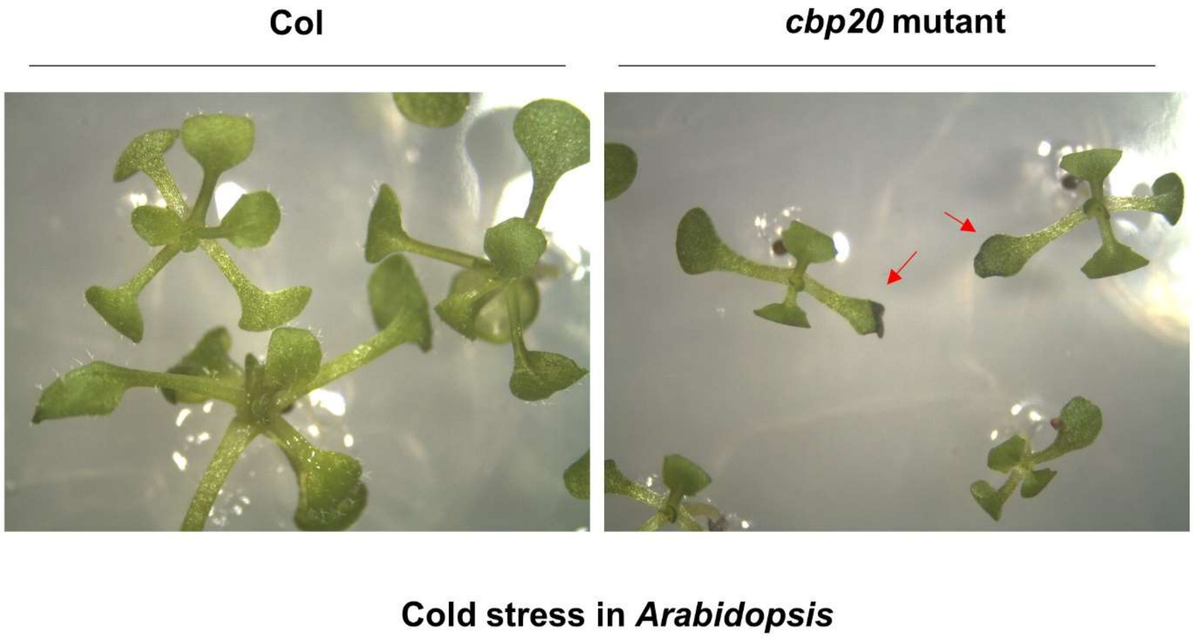

Figure S6. Phenotypes of wild type (Col-0) and *cbp20* mutant *Arabidopsis* in response to cold. The plants were treated at 4°C for 20 d under a 16-h light/8-h dark photoperiod condition.

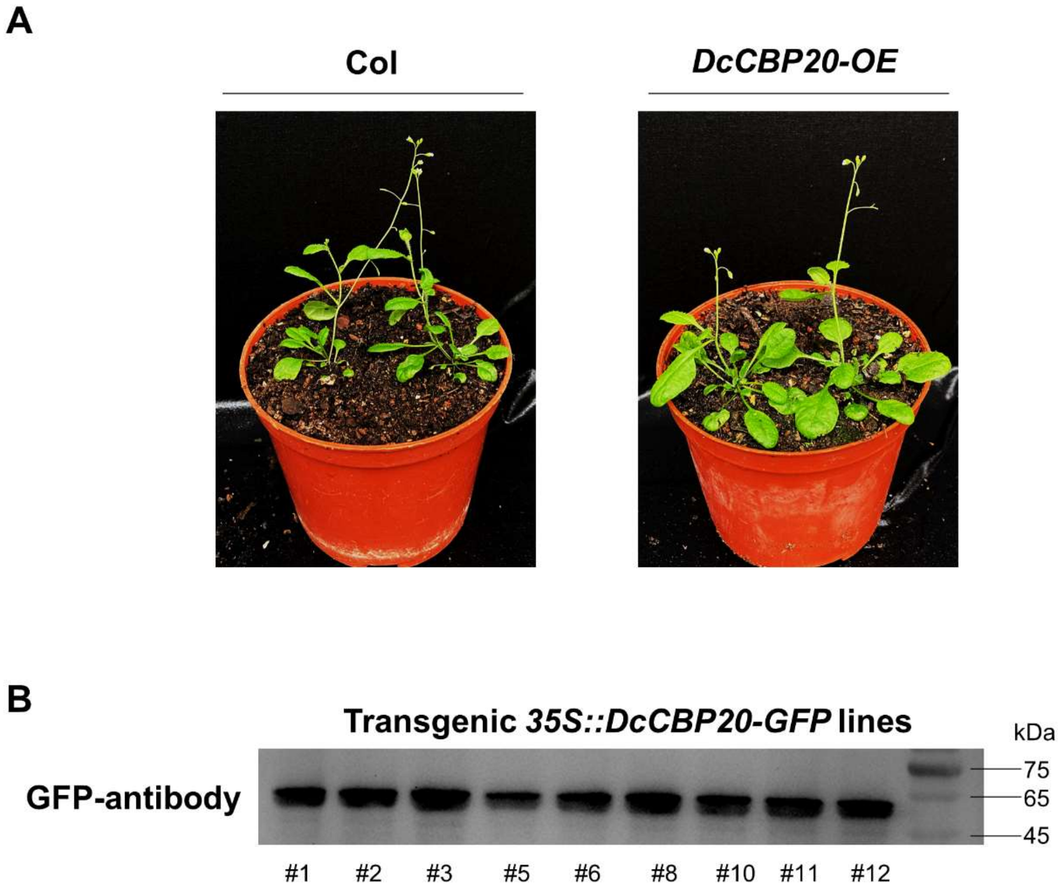

Figure S7. Generation of transgenic *Arabidopsis* overexpression of *DcCBP20*. **(A)** Phenotypes of the *DcCBP20* overexpressed *Arabidopsis*. **(B)** Identification of transgenic lines using western blot. The GFP antibody was used to detect the DcCBP20-GFP protein.

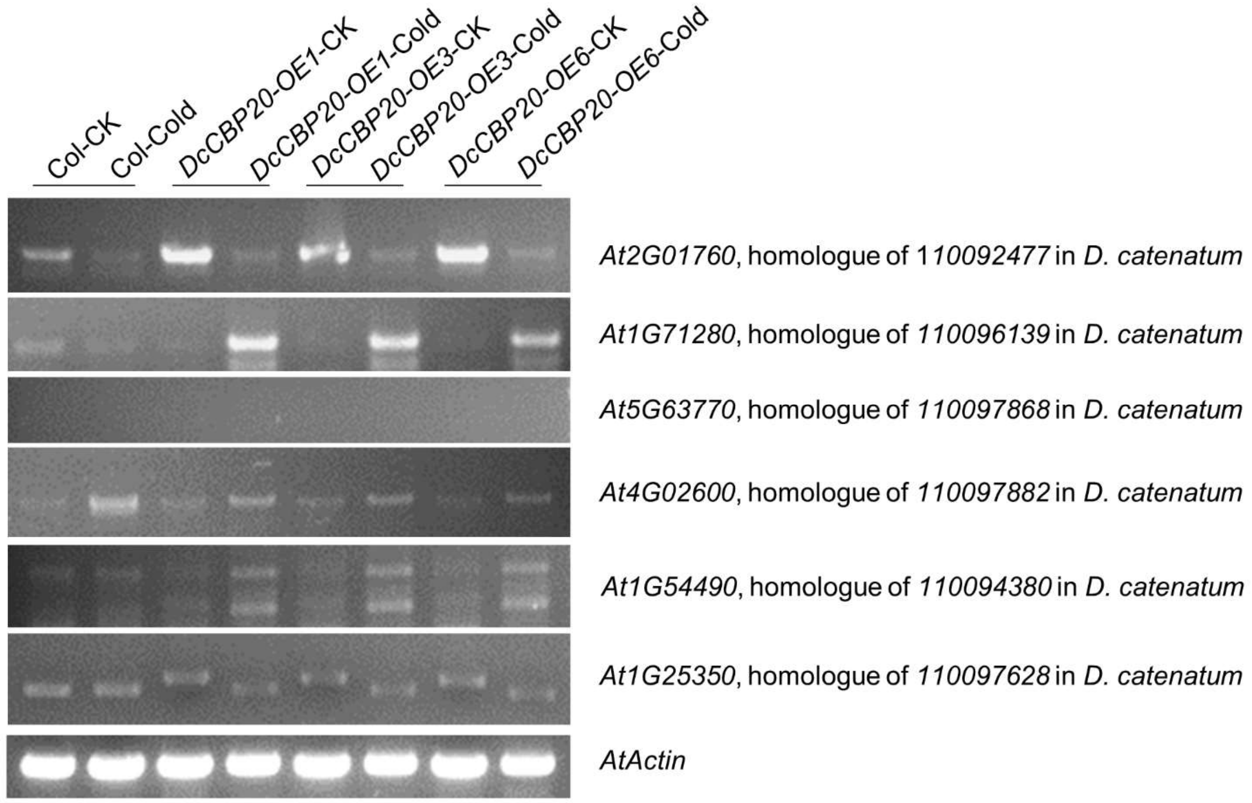

Figure S8. The splicing isoforms of the PCR products using specific primers in cDNA of *DcCBP20* transgenic *Arabidopsis* detected by RT–PCR. RT-PCR products were detected using 1.5% agarose gels.
